# The effect of bone marrow microenvironment on the functional properties of the therapeutic bone marrow-derived cells in patients with acute myocardial infarction

**DOI:** 10.1186/1479-5876-10-66

**Published:** 2012-04-02

**Authors:** Johanna A Miettinen, Riikka J Salonen, Kari Ylitalo, Matti Niemelä, Kari Kervinen, Marjaana Säily, Pirjo Koistinen, Eeva-Riitta Savolainen, Timo H Mäkikallio, Heikki V Huikuri, Petri Lehenkari

**Affiliations:** 1 Department of Internal Medicine, Institute of Clinical Medicine, University of Oulu, P.O. Box 5000, Kajaanintie 50, Oulu FIN-90014, Finland; 2 Department of Anatomy and Cell Biology, Institute of Biomedicine, University of Oulu, P.O. Box 5000, Kajaanintie 50, Oulu FIN-90014, Finland; 3 Department of Clinical Chemistry, Institute of Diagnostics, University of Oulu, P.O. Box 5000, Kajaanintie 50, Oulu FIN-90014, Finland

**Keywords:** Blood gas analysis, Bone marrow stem cells, Cell therapy, Mesenchymal stem cells, Myocardial infarction

## Abstract

**Background:**

Treatment of acute myocardial infarction with stem cell transplantation has achieved beneficial effects in many clinical trials. The bone marrow microenvironment of ST-elevation myocardial infarction (STEMI) patients has never been studied even though myocardial infarction is known to cause an imbalance in the acid-base status of these patients. The aim of this study was to assess if the blood gas levels in the bone marrow of STEMI patients affect the characteristics of the bone marrow cells (BMCs) and, furthermore, do they influence the change in cardiac function after autologous BMC transplantation. The arterial, venous and bone marrow blood gas concentrations were also compared.

**Methods:**

Blood gas analysis of the bone marrow aspirate and peripheral blood was performed for 27 STEMI patients receiving autologous stem cell therapy after percutaneous coronary intervention. Cells from the bone marrow aspirate were further cultured and the bone marrow mesenchymal stem cell (MSC) proliferation rate was determined by MTT assay and the MSC osteogenic differentiation capacity by alkaline phosphatase (ALP) activity assay. All the patients underwent a 2D-echocardiography at baseline and 4 months after STEMI.

**Results:**

As expected, the levels of pO_2_, pCO_2_, base excess and HCO_3 _were similar in venous blood and bone marrow. Surprisingly, bone marrow showed significantly lower pH and Na^+ ^and elevated K^+ ^levels compared to arterial and venous blood. There was a positive correlation between the bone marrow pCO_2 _and HCO_3 _levels and MSC osteogenic differentiation capacity. In contrast, bone marrow pCO_2 _and HCO_3 _levels displayed a negative correlation with the proliferation rate of MSCs. Patients with the HCO_3 _level below the median value exhibited a more marked change in LVEF after BMC treatment than patients with HCO_3 _level above the median (11.13 ± 8.07% vs. 2.67 ± 11.89%, P = 0.014).

**Conclusions:**

Low bone marrow pCO_2 _and HCO_3 _levels may represent the optimal environment for BMCs in terms of their efficacy in autologous stem cell therapy in STEMI patients.

## Background

Acute myocardial infarction (AMI) is the major cause of congestive heart failure and subsequent mortality in the developed countries. Despite the major advances in treatment methods, myocardial infarction usually causes irreversible damage to heart muscle. Cell therapy based on autologous stem cell transplantation and potential myocardial regeneration has been the focus of many clinical trials for more than a decade. These trials have yielded contradictory results which have been proposed to be attributable to the heterogeneity of the study designs [1]. A recent experimental study showed that donor myocardial infarction impaired the therapeutic potential of bone marrow cells in mice and it was hypothesized that this might explain why human cell therapy trials have not matched the success achieved in rodent experiments [2]. Moreover, it has not been previously studied if the bone marrow microenvironment of patients with ST-elevation myocardial infarction (STEMI) can affect the functionality of the cells used in autologous cell therapy. Myocardial infarction has been shown to result in metabolic acidosis [3] and base excess in the venous blood has proved to be an independent predictor for intra-intensive care unit mortality of STEMI patients [4]. Since the blood gases measured from venous blood have been shown to correlate with bone marrow measurements [5], the acid-base status of STEMI patients likely affects also the microenvironment of bone marrow. After a careful literature review, we were not able to find any previous data on bone marrow physiological conditions in adults. Thus, the aim of the present study was to assess if the blood gas or electrolyte levels in the bone marrow of STEMI patients can influence the characteristics of the bone marrow cells and, subsequently, the success of autologous stem cell transplantation. We also compared arterial, venous and bone marrow blood gas and electrolyte concentrations in patients with STEMI. This study is part of a pilot study (FINCELL II), which followed the original FINCELL (FINnish stem CELL) trial [6].

## Methods

We examined a consecutive series of STEMI patients who were admitted to the University Hospital of Oulu, Finland, or to the central hospitals of Kajaani, Rovaniemi or Kemi, Finland, between April 2008 and August 2009. Inclusion criteria were age 18-79 years, acute STEMI and depressed left ventricular function (LVEF < 55%) measured by echocardiography 2-5 days after STEMI. Exclusion criteria were unwillingness or inability to provide informed consent, NYHA class IV, psychological or physiological unsuitability for participation in the study, inaccessibility for follow-up due to geographical or other reasons, severe leukopenia or thrombocytopenia, hepatic or renal dysfunction or evidence for malignant disease. Patient characteristics are shown in Table 1. Written informed consent was obtained from patients within 5 days after STEMI. The study protocol conformed to the Declaration of Helsinki and was approved by the Ethical Committee of the Northern Ostrobothnia Hospital District. The original FINCELL trial is registered at http://www.clinicaltrials.gov with registration number NCT00363324.

**Table 1:** Patients characteristics

	Low dose (5 × 10^7^), n = 6	High dose (7 × 10^8^), n = 21
Age (years)	56 ± 10	60 ± 9

Male sex (%)	100	95

Body mass index	28 ± 5	28 ± 3

Hypertension [n(%)]	2 (33)	5 (24)

Diabetes [n(%)]	0	1 (5)

TnI max (μg/L)	52 ± 47	51 ± 56

Infarct-related vessel [n(%)]		
LAD	3 (50)	6 (29)
CX	0	0
RCA	3 (50)	14 (67)
LOM I	0	1 (5)

LVEF at baseline (%)	47.4 ± 9.2	44.4 ± 6.3

LVEF at 4 months' follow-up (%)	50.5 ± 13.2	52.3 ± 11.6

TIMI flow before PCI [n(%)]		
0	2 (33)	4 (19)
1	1 (17)	2 (10)
2	1 (17)	3 (14)
3	1 (17)	9 (43)

TIMI flow after PCI [n(%)]		
1	0	0
2	1 (17)	2 (10)
3	4 (33)	17 (81)

%Stenosis of the infarct-related artery before PCI	95 ± 5	93 ± 7

Time from STEMI to BMC injection (h)	268 ± 162	214 ± 94

Number of injected BMCs		
Number of mononuclear cells (× 10^6^)	54 ± 22	701 ± 254
Number of CD34+ cells (× 10^6^)	0.75 ± 0.46	8.9 ± 5.1

Adenosine infusion [n(%)]	0	9 (43)

Medication at discharge [n(%)]		
ASA	6 (100)	20 (95)
Clopidogrel	6 (100)	21 (100)
Beta-blocker	6 (100)	21 (100)
ACE inhibitor/AT II receptor blocker	6 (100)	21 (100)
Statin	6 (100)	18 (86)
Diuretic	2 (33)	9 (43)

Values are means ± SD. LAD, left anterior descending coronary artery; CX, circumflex coronary artery; RCA, right coronary artery; LOM, left obtuse marginal coronary artery; LVEF, left ventricular ejection fraction; PCI, percutaneous coronary intervention; STEMI, ST-elevation myocardial infarction; BMC, bone marrow stem cell, ASA, acetylsalicylic acid.

### Study design

The day of acute STEMI was defined as day 0. On days 1-5, patients were randomly assigned in a double-blinded fashion, either to the group receiving the small cell dose (10 ml, mean cell count 5 × 10^7^, n = 6) or to the group receiving the high cell dose (160 ml, mean cell count 7 × 10^8^, n = 21). The patients and investigators performing the cell injection procedures and analysis of data were unaware of the randomization throughout the study. Bone marrow aspiration, collection and preparation of cells were performed in the morning preceding the intracoronary injection of BMCs, which was performed 4-14 days post-STEMI.

### Cell preparation, administration and culturing

BMCs were collected in the morning of the cell administration day under local anesthesia and sterile conditions. 0.5-1 ml sample was aspirated from one-two iliac crest into a RAPIDLyte^® ^heparinized syringe and the blood gas and electrolyte levels were analysed using an i-STAT^® ^Point-of-Care System (Abbot Laboratories, Illinois, USA). 10 ml or 160 ml of bone marrow was aspirated and placed into tubes containing heparinized phosphate buffered saline (PBS; Gibco, Paisley, UK). The bone marrow cells were then transported to a culture laboratory dedicated to aseptic cell manipulation for autologous stem cell transplantation. The mononuclear cell fraction containing stem and progenitor cells was isolated by Ficoll density gradient centrifugation, washed twice with PBS and resuspended with 50% autologous serum in heparinized saline or with heparinized saline alone (total volume of 10 ml). The cells were kept at +4°C until use. The BMC separation procedure took approximately 3 hours and the cells were administered within 24 hours to the target coronary artery. One aliquot of the cell sample was subjected to quality-control procedures, i.e. microbial culture for sterility and flow cytometer analysis for CD34+ cell counting and determination of cell viability in the accredited laboratory of the Oulu University Hospital, which is subjected to both outside and inside quality control. The validity of the cell preparation system was assessed as described previously [6]. One aliquot of the BMC fraction was plated into 25 cm^2 ^tissue culture flask and cultured in 5 ml medium containing alpha MEM (Gibco) buffered with 20 mM HEPES (Gibco) and containing 10% heat-inactivated fetal calf serum (FCS; Bioclear, Netherlands), 100 U/ml penicillin, 0.1 mg/ml streptomycin, 2 mM L-glutamine (Gibco). Cells were cultured at +37°C in 5% CO_2 _and 95% air. After one day, the medium was changed and non-attached cells were washed away. The attached cells (mesenchymal stem cells, MSCs) were cultured in a flask and the medium was replaced two times per week until near-confluence. The cells were passed two times before the analyses. In the cell counting analyses, the cells were washed with PBS and adherent cells detached using trypsin-EDTA solution (Gibco).

### Flow cytometric analysis of cell surface antigens

Flow cytometric analysis of cell surface antigens was performed as previously described [7]. The minimum criteria panel of cell surface markers for hMSCs proposed by International Society for Cellular Therapy [8] was evaluated from all MSC lines analyzed, and each of the lines met the criteria. The percentage values of positive MSC markers were CD90 99.81 ± 0.23%, CD105 99.58 ± 0.37%, CD73 99.66 ± 0.28% and HLA-ABC 97.58 ± 3.06%. The percentage value of negative markers (CD14, CD34, CD45, CD19 and HLA-DR) was 1.80 ± 0.58%. Thus, the marker analysis showed that the cell cultures contained almost purely MSCs.

### MTT proliferation assay

MSCs were cultured in 96-well plates in six replicates. 500 cells/well were plated and half of the medium was changed twice a week. Cell proliferation was measured by MTT (3-(4,5-Dimethylthiazol-2-yl)-2,5-diphenyltetrazolium bromide) assay after 1, 4, 7, and 14 days culture. The medium was removed and MTT reagent (Sigma-Aldrich, St. Louis, MO, USA) (0,5 mg/ml in medium) was added to the cells and incubated for 2 h at +37°C in 5% CO_2 _and 95% air. After incubation, the MTT solution was removed and 100 μl/well of dimethyl sulfoxide (DMSO; Sigma-Aldrich) was added. The absorbance of the reduced form of MTT was measured at 550 nm and 650 nm (background) in a plate reader (Victor 2, Wallac Oy, Turku, Finland).

### Alkaline phosphatase activity assay

Human MSCs were seeded at 10000 cells/well into 24-well plates in four replicate wells and were cultured with the medium described above and another four replicates in a medium containing also 100 mM dexamethasone (Sigma, St. Louis, MO, USA), 10 mM β-glycerol (Sigma) and 0.05 mM ascorbic acid (Sigma). After 3 weeks, cultured cells were assayed in the following way: the assay buffer containing 0.1% Triton X-100, pH 7.6, was added to each well, and the plates were frozen. After thawing, alkaline phosphatase (ALP) activity was determined using 0.1 mM 4-p-nitrophenylphosphate (Sigma-Aldrich) as the substrate and absorbance was read at 405 nm in a plate reader (Victor 2, Wallac Oy, Turku, Finland). Each sample was measured in duplicate. The protein content of the wells was determined by the BIO-RAD Protein Assay (Bio-Rad Laboratories, Richmond, California, USA). The enzyme activities were expressed as units/mg protein.

### Blood sampling, blood gas and biochemical laboratory analyses

All the patients underwent venous and arterial blood sampling on the day of cell transplantation. pH, pO_2_, pCO_2_, HCO_3_, BE, Na^+ ^and K^+ ^levels were measured in the central laboratory of Oulu University Hospital.

### Measurement of left ventricular ejection fraction

A 2-dimensional echocardiogram was performed 2-5 days after STEMI and at 4 months after STEMI. The LVEF was measured by an experienced investigator in the core laboratory, unaware of the patient's treatment assignment, using the technique described previously [9].

### Statistical analysis

Variables are expressed as means ± SD, or medians with interquartile range with skewed data. Paired samples *t*-test was used to determine the statistical significance of the difference in laboratory values between bone marrow and arterial/venous blood. Correlation analyses between bone marrow and arterial/venous blood laboratory values were conducted using paired samples correlation. In other analyses, Pearson's correlation or Spearman's correlation with skewed data was used. Analysis-of-variance (ANOVA) was used in the between-group comparisons. The variance analyses of the LVEF change in HCO_3_, BE and potassium subgroups (above/below median) were corrected with the cell dose group (low/high dose). All P values are two-tailed and statistical significance was set at P < 0.05. Analyses were performed with SPSS software, version 14.0.

## Results

### Blood gas and electrolyte analyses of bone marrow, venous and arterial blood

**Figure 1:**
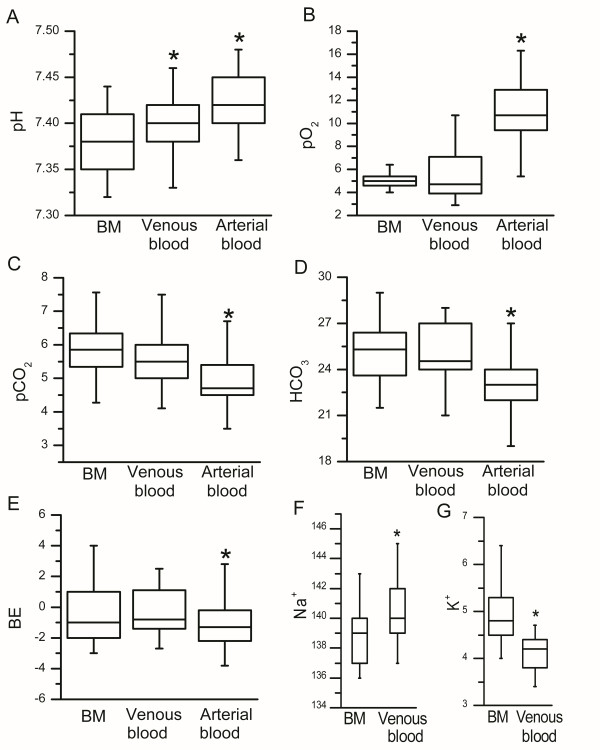
**The comparison of (A) pH, (B) PO_2_, (C) PCO_2_, (D) HCO_3_, (E) BE, (F) natrium, and (G) potassium levels between bone marrow (BM), venous and arterial blood**. P < 0.05 for difference compared to bone marrow.

The levels of blood gases (pO_2_, pCO_2_), serum bicarbonate (HCO_3_), base excess (BE), pH and electrolytes sodium (Na^+^) and potassium (K^+^) were measured from venous, arterial and bone marrow blood samples (Table 2, Figure 1). All laboratory values measured differed significantly between bone marrow aspirate and arterial blood. In addition, a significant difference was detected between bone marrow aspirate and venous blood in pH, sodium and potassium levels (Table 2). The levels of pO_2_, pCO_2_, BE and HCO_3 _were similar in venous blood and bone marrow.

**Table 2:** Laboratory analyses of bone marrow, venous and arterial blood

Laboratory variable	Bone marrow	Venous blood	Arterial blood	P value for paired difference (BM vs. vein)	P value for paired difference (BM vs. artery)
pH	7.38 ± 0.036	7.40 ± 0.031	7.42 ± 0.048	0.028	0.002

pO_2 _(kPa)	5.11 ± 0.68	5.63 ± 2.17	11.15 ± 2.79	0.26	< 0.001

pCO_2 _(kPa)	5.78 ± 0.81	5.62 ± 0.87	4.93 ± 0.84	0.27	< 0.001

HCO_3 _(mmol/L)	25.43 ± 2.83	25.25 ± 2.85	22.92 ± 1.86	0.66	< 0.001

BE (mmol/L)	-0.042 ± 2.46	0.20 ± 2.27	-1.09 ± 1.57	0.56	0.030

Na^+ ^(mmol/L)	138.96 ± 1.83	140.29 ± 2.26	n.a.	0.015	-

K^+ ^(mmol/L)	4.96 ± 0.64	4.06 ± 0.37	n.a.	0.000	-

Values are means ± standard deviation. BE = base excess, BM = bone marrow

### Association of bone marrow blood gas and electrolyte levels with the functional properties of bone marrow cells

**Figure 2:**
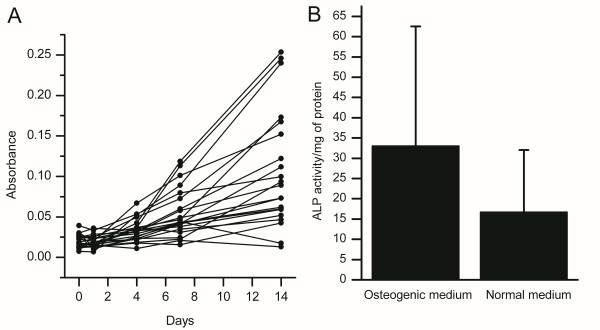
**Tests for bone marrow-derived mesenchymal stem cell (MSC) proliferation and differentiation**. **(A) MTT proliferation assay**. (B) Alkaline phosphatase (ALP) activity test of MSCs in normal growth medium and osteogenic differentiation medium.

The bone marrow mesenchymal stem cell (MSC) proliferation rate was determined by MTT assay (absorbance/time in days) (Figure 2A). The absorbance reading was plotted against time and the slope of the line was used as the measure of cell proliferation (average slope of all measurements 0.0076 ± 0.0086). The MSC differentiation capacity was determined by ALP activity assay (u/mg of protein in basic/differentiation medium) (Figure 2B). The associations of bone marrow blood gas and electrolyte levels with BM cell viability (87.9 ± 8.7%), MSC proliferation rate and the differentiation capacity of MSCs with bone marrow blood gas and electrolyte levels are presented in Table 3. Bone marrow pCO_2 _and HCO_3 _levels exhibited a negative correlation with the proliferation rate of mesenchymal stem cells. In contrast, there was a positive correlation between the bone marrow pCO_2 _and HCO_3 _levels and MSC ALP activity. This can be interpreted as better osteoblast differentiation capacity of MSCs when bone marrow pCO_2 _and HCO_3 _levels are high. However, none of the biochemical laboratory values measured was associated with the viability of cells in the bone marrow aspirate.

**Table 3:** Association of bone marrow blood gas and electrolyte levels with functional properties of bone marrow cells

Laboratory variable	CD34+ cell viability		MSC proliferation rate		MSC osteoblast differentiation	
	**Correlation coefficient**	**P-value**	**Correlation coefficient**	**P-value**	**Correlation coefficient**	**P-value**

pH	0.093	0.67	0.23	0.31	-0.41	0.095

pO_2 _(kPa)	0.009	0.97	0.16	0.48	-0.040	0.88

pCO_2 _(kPa)	0.002	0.99	-0.50	0.023	0.57	0.013

HCO_3 _(mmol/L)	0.085	0.69	-0.43	0.052	0.48	0.043

BE (mmol/L)	0.083	0.70	-0.41	0.066	0.34	0.17

Na^+ ^(mmol/L)	0.10	0.64	0.27	0.24	-0.015	0.95

K^+ ^(mmol/L)	0.147	0.50	0.012	0.96	0.037	0.88

BM = bone marrow, MSC = mesenchymal stem cell

### Association of bone marrow blood gas and electrolyte levels with the functional recovery of the heart after cell therapy

The association of the functional recovery of the heart after cell therapy with bone marrow blood gas and electrolyte levels was evaluated by studying the correlations of the blood gases with the change of LVEF between baseline and the 4 months' follow-up. There was a trend towards a negative correlation between LVEF change and bone marrow HCO_3 _level as well as the BE level and a positive correlation between LVEF change and potassium level, but these associations did not reach statistical significance (Table 4).

**Table 4:** Association of bone marrow blood gas and electrolyte levels with the change of LVEF after cell therapy

Laboratory variable	Change of LVEF (%-points)	
	
	Correlation coefficient	P-value
pH	0.063	0.77

pO_2 _(kPa)	0.16	0.45

pCO_2 _(kPa)	-0.31	0.13

HCO_3 _(mmol/L)	-0.37	0.070

BE (mmol/L)	-0.36	0.082

Na^+ ^(mmol/L)	-0.26	0.23

K^+ ^(mmol/L)	0.39	0.059

**Figure 3:**
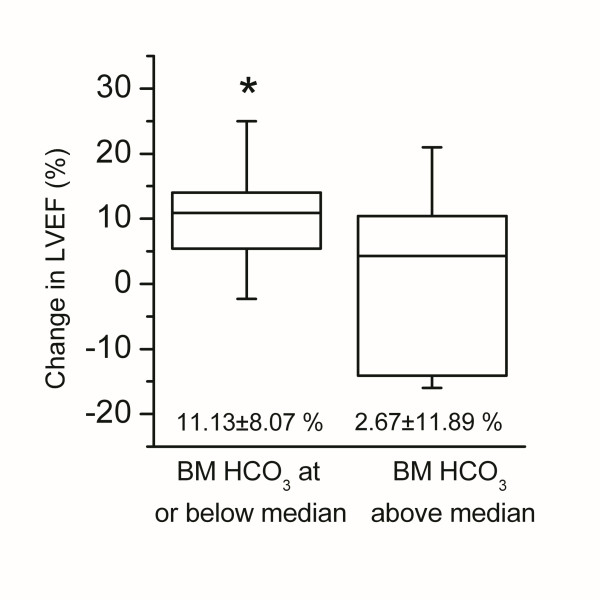
**Interaction between bone marrow (BM) HCO_3 _level and the absolute change in left ventricular ejection fraction (LVEF) shown as box (25-75% percentiles) and whiskers (range) with median (-)**.* P < 0.05 for difference between the groups.

In a further analysis, the patient population was divided into two subgroups according to their HCO_3_, BE and potassium values, respectively, and the cell dose group was used as a covariate in the analyses. Patients with the HCO_3 _level below the median value exhibited a more marked change in LVEF after stem cell treatment than patients with the HCO_3 _level above the median (11.13 ± 8.07% vs. 2.67 ± 11.89%, P = 0.014) (Figure 3). No significant differences were found in the LVEF change between BE or potassium subgroups (data not shown). In addition, the cell dose did not have a significant effect on the LVEF change (p = 0.067).

In an attempt to clarify the individual differences in the levels of bone marrow blood gases, the associations of several other parameters (e.g. age, sex, body mass index, hypertension, hypercholesterolemia, diabetes, heart failure, severity of CAD, medication at discharge) with the bone marrow blood gas values were tested. The bone marrow BE and HCO_3 _levels were found to be higher in patients using diuretic drugs at the time of hospital discharge than in patients with no diuretic medication (BE 1.10 ± 2.81 vs. -0.87 ± 1.81 mmol/L, P = 0.043 and HCO_3 _26.69 ± 3.46 vs. 24.54 ± 1.89 mmol/L, P = 0.056, respectively). In addition, the patients using diuretics experienced a smaller improvement in the LVEF than patients without diuretic medication (1.81 ± 12.42 vs. 10.24 ± 7.41%-units, respectively, P = 0.036). No significant associations were found between any other patient characteristics and the bone marrow blood gases (P = NS in each analysis, data not shown).

## Discussion

The results of the present study demonstrate that there is a significant difference in blood gas and electrolyte levels between bone marrow and arterial blood in patients with STEMI. In addition, a significant difference was found between bone marrow aspirate and venous blood in pH, sodium and potassium levels. In contrast, the levels of pO_2_, pCO_2_, BE and HCO_3 _were similar in venous blood and bone marrow. In a further blood gas analysis, the proliferation rate of bone marrow-derived MSCs was associated with the pCO_2 _and HCO_3 _levels in the bone marrow. The MSC proliferation was more efficient when bone marrow pCO_2 _and HCO_3 _levels were low. It was also shown that a low HCO_3 _level in bone marrow was associated with better functional recovery of the heart after myocardial infarction treated with intracoronary injection of autologous BM cells. Patients with an HCO_3 _level below the median value experienced a more marked improvement of LVEF than patients with the HCO_3 _level above the median value. In contrast, the osteogenic differentiation potential of the BM-derived MSCs was shown to be better when bone marrow pCO_2 _and HCO_3 _levels were high.

One objective of this study was to compare arterial, venous and bone marrow blood gas and electrolyte concentrations in STEMI patients. As far as we are aware, only one previous study has assessed the levels of blood gases and electrolytes in bone marrow and venous blood in humans [5]. Grisham et al. studied children with acute leukemia or other hematological disorders. In those patients, a significant correlation was found between venous and bone marrow samples for pH, pCO_2_, HCO_3_, BE and sodium concentration, but no predictable relationship was observed for pO_2 _and potassium concentration. In the present study, pCO_2_, BE and HCO_3 _also correlated between bone marrow and venous blood as well as bone marrow and arterial blood. However, pH, sodium and potassium levels differed significantly between venous blood and bone marrow. In the study of Grisham et al. the actual differences between bone marrow and venous blood were not assessed. However, they also noticed that the potassium levels differed significantly between bone marrow and venous blood. They considered that this could be due to the traumatic nature of specimen collection and the high negative pressure generated in aspirating marrow evoking hemolysis [5]. A portion of the cells inevitably break during the bone marrow aspiration but we think that the cell breakage does not fully explain the difference in potassium concentration between bone marrow and venous blood because we found a difference also in the sodium concentration. Instead, it may be a true finding being related to the different activity of sodium-potassium pump.

Bicarbonate is alkaline, and it is one of the components that maintain acid-base homeostasis in the body. Most of the CO_2 _in the body is converted into carbonic acid (H_2_CO_3_), which rapidly forms bicarbonate (HCO_3_^-^). The pH in the body depends on the pCO_2 _and the level of HCO_3_^-^. In this study MSC proliferation was found to be more efficient when the bone marrow aspirate pCO_2 _and HCO_3 _levels were low. The pH in the bone marrow was not directly associated to the proliferation rate but this may be due to other compensatory buffering mechanisms, such as hemoglobin, which is known to stabilize the pH value. It is also known that active inflammation creates an acidic environment [10]. In the present study, the levels of pCO_2 _and HCO_3 _displayed a nonsignificant negative correlation with bone marrow interleukin (IL)-6 levels (data not shown). Thus, the levels of pCO_2 _and HCO_3 _may have declined in an attempt to buffer the acidosis caused by inflammatory processes. We have previously shown that inflammation (mediated by tumour necrosis factor alpha) enhances the proliferation of MSCs and activates many immunosuppressive pathways *in vitro *[7]. Thus, if inflammation is more extensive in the bone marrow of the patients with lower levels of pCO_2 _and HCO_3_, it can be postulated to have an influence on the resident MSCs and enhance also their *in vitro *proliferation. On the contrary, the osteogenic differentiation potential of the BM cells was found to be better when bone marrow pCO_2 _and HCO_3 _levels were high. This result is in line with previous studies which have shown that acidic environment impairs osteoblast function,[11,12] reduces osteoblast alkaline phosphatase (ALP) activity [13,14] and inhibits osteogenic differentiation of MSCs [14,15].

Several previous trials have shown that the number of bone marrow CD34+ cells does not correlate with the change of LVEF after cell transplantation [16-19]. In the present study, the viability of CD34+ cells was not associated with the pCO_2 _and HCO_3 _levels. In addition, the observation that low bone marrow pCO_2 _and HCO_3 _is beneficial for BM-derived MSC proliferation but not for the osteoblast differentiation suggests that maintaining the stemness of MSCs or some other cell lineage is predominant in this kind of environment. It has been previously shown that hMSCs cultured in hypoxic conditions (2% oxygen) display significantly improved expansion characteristics while maintaining their multi-lineage potential [20]. On the other hand, hypoxia as such can lead to unfavorable effects in the cultured cells, such as chromosomal aberrations [21]. Obviously, one of the most important functions of the bone marrow microenvironment is to support the stemness of the resident BMCs and protect the infinitely dividing cell population from oxygen derived free radicals that could theoretically promote the cancer propagation of these cells. It has also been shown that bone marrow contains many different microenvironments in which MSCs are the prominent cell components; these cells play many important roles including controlling hematopoiesis [22,23]. Based on our results, it seems that the optimal environment for BM cells in this respect is associated with a low bone marrow pCO_2 _value and reduced HCO_3 _levels which support the stemness of BM cells making them more efficient for cell therapy use and also more capable of undergoing intrinsic repair mechanisms. However, the results of this study are still preliminary findings and the importance of bone marrow microenvironment on MSC functionality after two MSC passages in culture media with 5% CO_2 _and 95% air, which are completely different conditions compared to the bone marrow of STEMI patients, remain to be elucidated. Further tests in cell cultures are currently ongoing and will later provide us more insights about the effect of different environmental factors of the bone marrow on the functionality of the BM cells.

As far as we are aware, this is the first study to assess the effects of the metabolic state of bone marrow on the functional capacity of the bone marrow-derived stem cells in STEMI patients. It has been previously shown that age, risk factors for coronary artery disease, diabetes and heart failure diminish the capacity of the blood-derived circulating cells to contribute to the functional repair compared to healthy controls [24-26]. In addition, when the number and functionality of bone marrow-derived cells have been analyzed, the presence of heart failure also has been shown to affect these cells. BMCs isolated from bone marrow aspirates of patients with ischemic heart failure have been less effective in achieving a recovery of blood flow after hind limb ischemia compared to cells from healthy controls [27]. The comparison of the composition and function of BMCs has demonstrated that the number of granulocyte-macrophage colony-forming units (GM-CFUs) is significantly lower and the migratory capacity of the cells is reduced in patients with ischemic heart failure as compared to healthy controls [27,28]. Moreover, chronic heart failure has been shown to be an independent predictor of bone marrow cell impairment, whereas the cardiovascular risk factors have not been statistically predictive [28]. We have also earlier shown that the energy metabolism of bone marrow mesenchymal cells is different in aged (> 50 years old) patients compared to young (< 18 years) and umbilical cord blood mesenchymal stem cells [29]. In the present study, the age of the patients was not associated with the parameters describing the metabolic state of bone marrow or the viability of BMCs. Heart failure, hypercholesterolemia, diabetes or hypertension were not associated to these parameters either. However, this may be due to the small patient number in each disease group. Further studies with larger patient populations are needed to clarify the association between these diseases and the bone marrow metabolic state. There were also attempts to collect samples from healthy hip fracture patients and patients undergoing spinal surgery to compare their bone marrow blood gas levels with STEMI patients. However, there were serious technical difficulties in the measurement of the thick bone marrow sample taken from the open bone and also arterial blood 'contamination'. Thus, the comparison of blood gas values between healthy subjects and patients with myocardial infarction remains to be elucidated in future studies.

The LVEF change between baseline and follow-up visit has been the most widely used endpoint to evaluate the effectiveness of stem cell transplantation after myocardial infarction [1]. In the present study, patients with the bone marrow HCO_3 _level below the median value experienced a more marked improvement of LVEF after stem cell treatment than their counterparts with an HCO_3 _level above the median value. This clinical trial was designed to be a dose-response study but no difference in the LVEF recovery could be observed between the groups receiving low or high dose of BMCs in this pilot phase. Thus, it is not likely that the differences in the cell doses would have affected the EF results of the present substudy. However, because of the low patient number, the results of this study have to be interpreted as descriptive rather than confirmatory when considering the importance of the blood gas values of the bone marrow on the clinical outcome of BMC treated STEMI patients. More studies with higher patient number are needed to confirm these preliminary findings.

**Figure 4:**
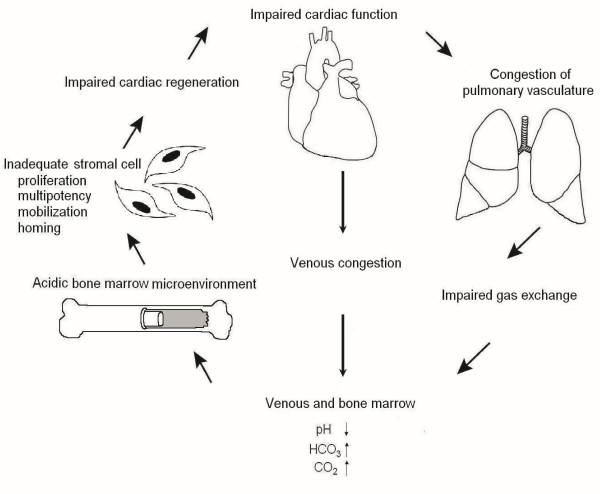
**Association between heart, pulmonary vasculature, peripheral circulation and bone marrow after an ischemic event**.

The bone marrow BE and HCO_3 _levels were found to be significantly higher in patients using diuretic drugs at the time of hospital discharge than in patients with no diuretic medication. The patients using diuretics also experienced less extensive improvement in the LVEF than the patients with no diuretic medication. The use of diuretic drugs is usually associated with post-STEMI hypertension or to venous or pulmonary congestion, and it is these conditions that most likely are affecting the blood gas values rather than the medication itself. Based on the results of the present study, we have created a hypothetical model describing the associations between heart, pulmonary vasculature, peripheral circulation and bone marrow after an ischemic event (Figure 4). In this model, the impaired cardiac function leads to venous or pulmonary congestion making bone marrow more acidic and, as a buffering effect, there are elevations in the pCO_2 _and HCO_3 _concentrations in the peripheral circulation as well as in the bone marrow. The acidic bone marrow microenvironment also impairs the function of the resident stromal cells which leads to impaired cardiac regeneration after myocardial infarction. The course of events creates a vicious cycle causing even more impairment of cardiac function. This idea is supported by a previous experimental work which showed that donor myocardial infarction impaired the therapeutic potential of bone marrow cells in mice [2]. However, based on the results of the present study, we cannot be sure if it is the change in cardiac, venous or pulmonary function that triggers this process. Thus, further studies with a higher number of patients will be needed to clarify the pathways which are responsible for the individual differences in acid-base status and the metabolic state of the bone marrow in STEMI patients. We believe that this study suggests that we should not ignore the role of bone marrow physiology as a regulator of BMC-mediated tissue repair, both therapeutic and spontaneous.

## Conclusions

There is a significant difference in blood gas and electrolyte levels between bone marrow and arterial blood in patients with STEMI. There are also significant differences in the pH, sodium and potassium levels between bone marrow aspirate and venous blood. Even though bone marrow acid-base status and blood gas levels resemble more closely venous blood than its arterial counterpart, the bone marrow seems to form a unique compartment for maintenance and storage of stromal cells, similarly to that reported in the context of the hematopoietic stem cell niche. The proliferation rate of bone marrow-derived MSCs is more efficient when bone marrow pCO_2 _and HCO_3 _levels are low. In contrast, the osteogenic differentiation potential of the BM-derived MSCs is better when bone marrow pCO_2 _and HCO_3 _levels are high. The STEMI patients with lower HCO_3 _levels experience a more marked change in LVEF. Moreover, patients using diuretic drugs at the time of hospital discharge tended to have higher bone marrow pCO_2 _and HCO_3 _levels and these patients experienced a poorer improvement in the LVEF after stem cell therapy, most probably because of the underlying cardiac or venous congestion affecting also the bone marrow metabolic state. In this respect, low bone marrow pCO_2 _and HCO_3 _levels represent the optimal environment for BM cells in terms of their efficacy in autologous stem cell therapy in STEMI patients.

## Competing interests

The authors declare that they have no competing interests.

## Authors' contributions

JM participated in the design of the study, made laboratory analyses and cell culturing, acquired the data, analysed and interpreted the data, performed statistical analyses and drafted the manuscript.

RS made laboratory analyses and cell culturing, acquired the data, analysed and interpreted the data and helped to draft the manuscript. KY conceived and designed the study, chose and examined the patients, performed 2D-echocardiography and other medical interventions for the study patients, injected the cells, acquired the data and analysed and interpreted the data. MN and KK chose and examined the patients, performed 2D-echocardiography and other medical interventions for the study patients, injected the cells, acquired the data and analysed and interpreted the data. MS and PK conceived and designed the study, performed cell aspirations from the bone marrow, performed laboratory analyses, examined the patients, acquired the data, analysed and interpreted the data and helped to draft the manuscript. E-R.S conceived and designed the study, acquired the data, handled funding and supervision and helped to draft the manuscript. TM conceived and designed the study, chose and examined the patients, performed medical interventions for the study patients, acquired the data and handled supervision. HH conceived and designed the study, chose and examined the patients, performed medical interventions for the study patients, acquired the data, analyzed and interpreted the data, handled funding and supervision and helped to draft the manuscript. PL conceived and designed the study, acquired the data, analyzed and interpreted the data, handled funding and supervision and helped to draft the manuscript. All authors read and approved the final manuscript.
